# Ab-Externo AAV-Mediated Gene Delivery to the Suprachoroidal Space Using a 250 Micron Flexible Microcatheter

**DOI:** 10.1371/journal.pone.0017140

**Published:** 2011-02-11

**Authors:** Marc C. Peden, Jeff Min, Craig Meyers, Zachary Lukowski, Qiuhong Li, Sanford L. Boye, Monica Levine, William W. Hauswirth, Ramakrishna Ratnakaram, William Dawson, Wesley C. Smith, Mark B. Sherwood

**Affiliations:** Ophthalmology, University of Florida, Gainesville, Florida, United States of America; University of Kansas Medical Center, United States of America

## Abstract

**Background:**

The current method of delivering gene replacement to the posterior segment of the eye involves a three-port pars plana vitrectomy followed by injection of the agent through a 37-gauge cannula, which is potentially wrought with retinal complications. In this paper we investigate the safety and efficacy of delivering adeno-associated viral (AAV) vector to the suprachoroidal space using an ab externo approach that utilizes an illuminated microcatheter.

**Methods:**

6 New Zealand White rabbits and 2 Dutch Belted rabbits were used to evaluate the ab externo delivery method. sc-AAV5-smCBA-hGFP vector was delivered into the suprachoroidal space using an illuminated iTrackTM 250A microcatheter. Six weeks after surgery, the rabbits were sacrificed and their eyes evaluated for AAV transfection using immunofluorescent antibody staining of GFP.

**Results:**

Immunostaining of sectioned and whole-mounted eyes demonstrated robust transfection in all treated eyes, with no fluorescence in untreated control eyes. Transfection occurred diffusely and involved both the choroid and the retina. No apparent adverse effects caused by either the viral vector or the procedure itself could be seen either clinically or histologically.

**Conclusions:**

The ab externo method of delivery using a microcatheter was successful in safely and effectively delivering a gene therapy agent to the suprachoroidal space. This method presents a less invasive alternative to the current method of virally vectored gene delivery.

## Introduction

While the field of ophthalmology continues to be revolutionized by new pharmacologic and biologic treatments, techniques for the delivery of such therapies have only evolved minimally. Recently, promising advances have been made in the treatment of Leber’s congenital amaurosis (LCA)—a rare form of hereditary childhood blindness—through the use of ocular gene therapy. LCA2 is a form of recessively inherited retinal blindness caused by mutations in the RPE65 gene, which is expressed in retinal pigmented epithelium and codes for a 65-kD protein [1]–[3]. Without a functional RPE65 gene, rod and cone photoreceptor cells become deficient in 11-*cis* retinal and are unable to respond to light [1]. This results in impaired vision from birth and a deterioration of vision that results in total blindness by the third or fourth decade of life [1], [2].

Several human trials investigating the use of adeno-associated virus (AAV) as a vector for transferring wild-type RPE65 into the retina have demonstrated that such treatments can lead to marked visual improvements with minimal to no adverse effects [1]–[5]. Typically, the technique for delivering the vector consists of a three-port pars plana vitrectomy with subretinal injection of the viral complex through a 37-gauge cannula [1]–[3]. In trials conducted by Maguire, *et al.* and Simonelli, *et al.,* one of six and one of three patients respectively developed a macular hole in the post-operative period [2], [5]. In both cases, it was concluded that the macular hole was likely due to a pre-existing epiretinal membrane contraction that was stimulated by the surgical procedure itself rather than the vector [2], [5].

This post-operative finding highlights the potential benefits of ab-externo delivery of gene replacement and other potential therapeutic agents into the subretinal space. Using the rabbit as an animal model, we explore the possibility of delivering AAV vector to the suprachoroidal space using the iTrackTM 250A microcatheter. This microcatheter, which was originally designed for use in the Schlemm’s canal for the treatment of open-angle glaucoma [6], was later shown to be safe and effective in introducing drugs into the posterior segment of the eye [7]. Compared to a vitrectomy, this approach is a much quicker and less invasive method of delivering therapeutic agents into the eye. For the first time, our study investigates the safety and effectiveness of delivering a gene replacement vector through this method.

## Materials and Methods

### Study Design

Six adult New Zealand White rabbits and two Dutch Belted rabbits were used in this study. All animal experiments performed adhered to the ARVO Statement for the Use of Animals in Ophthalmic and Vision Research and were approved by the University of Florida’s Institutional Animal Care and Use Committee, study number 200801808.

The right eyes of the albino rabbits received vector while the left eyes were used as untreated controls. Between the two Dutch Belted rabbits, three pigmented eyes received vector while one was used as a control.

### Vector Delivery

All eyes were dilated and the rabbits were anesthetized with a 35 mg/kg ketamine (“Ketaject”, Phoenix, MO) and intramuscular 5 mg/kg xylazine (“Xyla-ject”, Phoenix, MO). A 4–5 mm superior limbal conjunctival peritomy was performed to expose bare sclera. A #75 Beaver™ blade (Becton Dickinson & Co., Franklin Lakes, NJ) was used to perform a 1–2 mm radial incision to expose bare choroid approximately 3–4 mm posterior to the limbus.

The iTrack^TM^ 250A microcatheter (provided courtesy of iScience Interventional, Menlo Park, CA) connected to the iLumin^TM^ laser-diode based micro-illumination system (iScience Interventional, Menlo Park, CA) was then introduced into the suprachoroidal space and advanced posteriorly toward the optic disc (Figs. S1 and S2). Using indirect ophthalmoscopy, the illuminated catheter tip was further manipulated and confirmed to be at the posterior pole or in the retinal periphery. 100 µl of sc-AAV5-smCBA-hGFP vector at a concentration of 4.5×10^13^ vector genomes/ml (produced as described by Zolotukhin, *et al.* [8]) was injected into the potential space. The microcatheter was extracted and the conjunctiva closed with 8-0 Vicryl suture (Ethicon Inc., Somerville, NJ).

The surgical eyes received ciprofloxacin drops at the end of the case and were examined by indirect ophthalmoscopy at 1, 3, and 6 weeks post-operatively.

### Histology

The rabbits were sacrificed at 6 weeks after surgery. The globes were removed and placed in 4% paraformaldehyde in phosphate-buffered saline (PBS) for 24 hrs and then incubated in 30% sucrose in PBS for cryoprotection. Whole mounts were prepared for all 4 pigmented rabbit eyes and 8 albino eyes (4 treated, 4 untreated). The remaining 4 albino eyes (2 treated, 2 untreated) were embedded in Optimum Cutting Temperature (O.C.T., Sakura Finetek USA, Torrance, CA) then frozen at −20°C and cryosectioned at 25 microns.

The sections were immunostained using a mouse anti-GFP primary antibody (courtesy of Dr. W. Clay Smith, University of Florida) and Alexa 488 fluorophore secondary antibody (Invitrogen, Eugene, OR). Fluorescence was visualized using scanning confocal microscopy.

## Results

In each of the treated eyes, we were able to effectively access and cannulate the suprachoroidal space. The correct location of the illuminated cannula tip was confirmed under direct visualization. A localized suprachoroidal detachment was observed after the administration of 100 µl of vector. Two rabbits demonstrated loss of retinal perfusion during administration indicative of increased intraocular pressure. This necessitated an anterior chamber paracentesis with rapid reestablishment of good perfusion. All rabbits tolerated the surgical procedure and anesthesia without any complication. Subsequent clinical examinations on post-operative day (POD) 1 demonstrated resolution of the suprachoroidal detachment without any evidence of retinal hemorrhage or damage. Further follow-up showed no evidence of retinal detachment, ocular infection, vitreous hemorrhage, or other adverse events.

**Figure 1:**
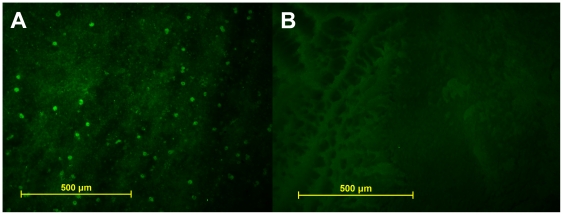
Fluorescent light micrograph of retinal whole mounts, oriented photoreceptors downward. A. Treated right eye of a New Zealand white rabbit demonstrates immunofluroescence; **B**. Non-treated left eye of the same rabbit demonstrates lack of immunofluorescence.

**Figure 2:**
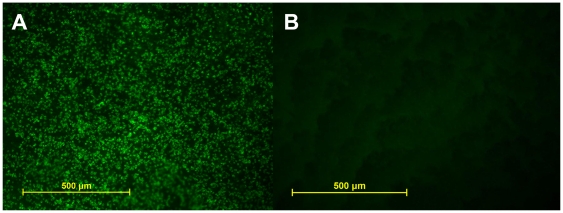
Fluorescent light micrographs of choroid in whole mount. A. Intense immunofluorescence seen in treated left eye from a Dutch Belted rabbit. **B**. Non-treated left eye showing lack of immunofluorescence.

**Figure 3:**
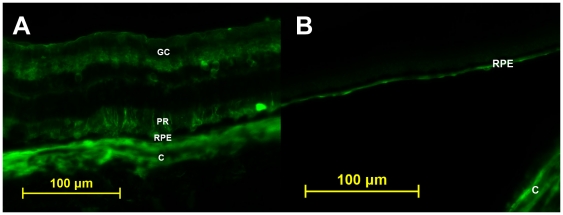
Fluorsecent light micrographs of retinal cross sections. **A**. Treated eye of a Dutch Belted rabbit demonstrating immunofluorescence likely occuring at the level of choroid (C), retinal pigmented epithelium (RPE), photoreceptors (PR), and retinal ganglion cells (GC). **B**. Untreated eye of a Dutch Belted rabbit with autofluorescence seen at the level of choroid (C) and retinal pigmented epithelium (RPE).

Six weeks following vector delivery, examination of whole-mounted treated eyes demonstrated robust transfection corresponding to the area of injection-induced suprachoroidal detachment (Fig. 1A). Examination of whole-mounted eyes confirmed an absence of GFP in controls (Fig. 1B). Efficacy of transfection for treated eyes appeared similar among all eyes by microscopic examination of the histological specimen in both NZW and Dutch Belted rabbits. Although direct staining of the retina and choroid was not performed, it is believed from the whole mounts (Fig. 2) and sections (Fig. 3) that GFP transfection occurred at the level of the choroid, retinal pigmented epithelium (RPE), photoreceptors, and retinal ganglion cells. No evidence of inflammation or tissue destruction was noted.

## Discussion

Considering the recent progress in the treatment of LCA2 and color blindness using ocular gene therapy [1]–[5], the successful delivery of gene-replacement and other therapeutic agents into the eye has become a potentially important treatment option for many retinal diseases. While the development of these therapeutic agents has progressed dramatically, the method for delivering these products has remained largely unchanged. The standard method of delivery—a three-port pars plana vitrectomy followed by a subretinal injection—carries a risk of surgical complications. Previous trials using this technique to deliver AAV by subretinal injection of virus following vitrectomy have demonstrated the possibility of creating a full-thickness macular hole [2], [5]. In addition, Hauswirth, *et al*. reported the presence of foveal thinning in one patient [3].

In our study, we were able to safely deliver AAV vector to the suprachoroidal space in the rabbit model, with no apparent adverse effects caused by either the viral vector or the procedure itself. We were also able to show that the vector successfully delivered the gene product into an injection-induced bleb within the retina and choroid. Compared to a standard three-port pars plana vitrectomy with a subretinal injection, the ab-externo method of vector delivery using the microcatheter is a much quicker and less invasive procedure with theoretically less risk of complications to the retina and surrounding tissues. As therapy moves towards treatment of patients at a younger age, the advantage of avoiding potential contact with the crystalline lens or disruption of the hyaloid face may result in this being a much safer and preferred method of delivery. As surgeons become more skilled in manipulating the microcatheter, it may be possible to deliver multiple, targeted treatments to specific areas of the retina without requiring multiple injections through the retina, as would be the case with the current vitrectomy technique. Additionally, even more specific targeting of cell types can be achieved by utilizing AAV serotypes with varying tissue tropism and/or tissue specific promoters [9], [10].

A number of potential future uses for this technique are currently being investigated. Recently, it has been shown that subretinal injections of AAV can deliver the L-opsin gene to the photoreceptor layer, restoring trichromatic vision in adult monkeys effected by red-green colorblindness [10]. However, in the aforementioned study, successful transfection was localized only to the areas around the injection sites. The microcatheter technique using the suprachoroidal space has the potential of being able to deliver virus at multiple sites through a single incision port in the eye, which may result in a greater overall area of transfection that would be beneficial in diseases involving the whole retina. Furthermore, AAV is being investigated as a potential delivery agent for anti-angiogenic therapy aimed at treating choroidal neovascularization [11].

We are currently investigating the effects of virus titer, serotype, capsid structure, and incubation period on the efficacy of retinal and choroidal transfection. Additionally, further investigation is needed to compare the efficacy of transfection between this method and the standard three-port vitrectomy and subretinal injection, as well as a more in-depth analysis of potential complications.

## Supporting Information

Figure S1 **Illuminated microcatheter tip in the posterior suprachoroidal space, visualized by indirect ophthalmoscopy.**
(TIF)

Figure S2 **Schematic of iScience 250A microcannula (top) and introduction of the microcatheter through the sclerotomy into the suprachoroidal space and advanced towards the optic disc (bottom).** Photographs courtesy of iScience Interventional.
(TIF)
