# Supplementary figures and images for: Ab-Externo AAV-Mediated Gene Delivery to the Suprachoroidal Space Using a 250 Micron Flexible Microcatheter
Source: PLoS One. 2011 Feb 11;6(2):e17140. doi: 10.1371/journal.pone.0017140 (PMC3037961; doi:10.1371/journal.pone.0017140)

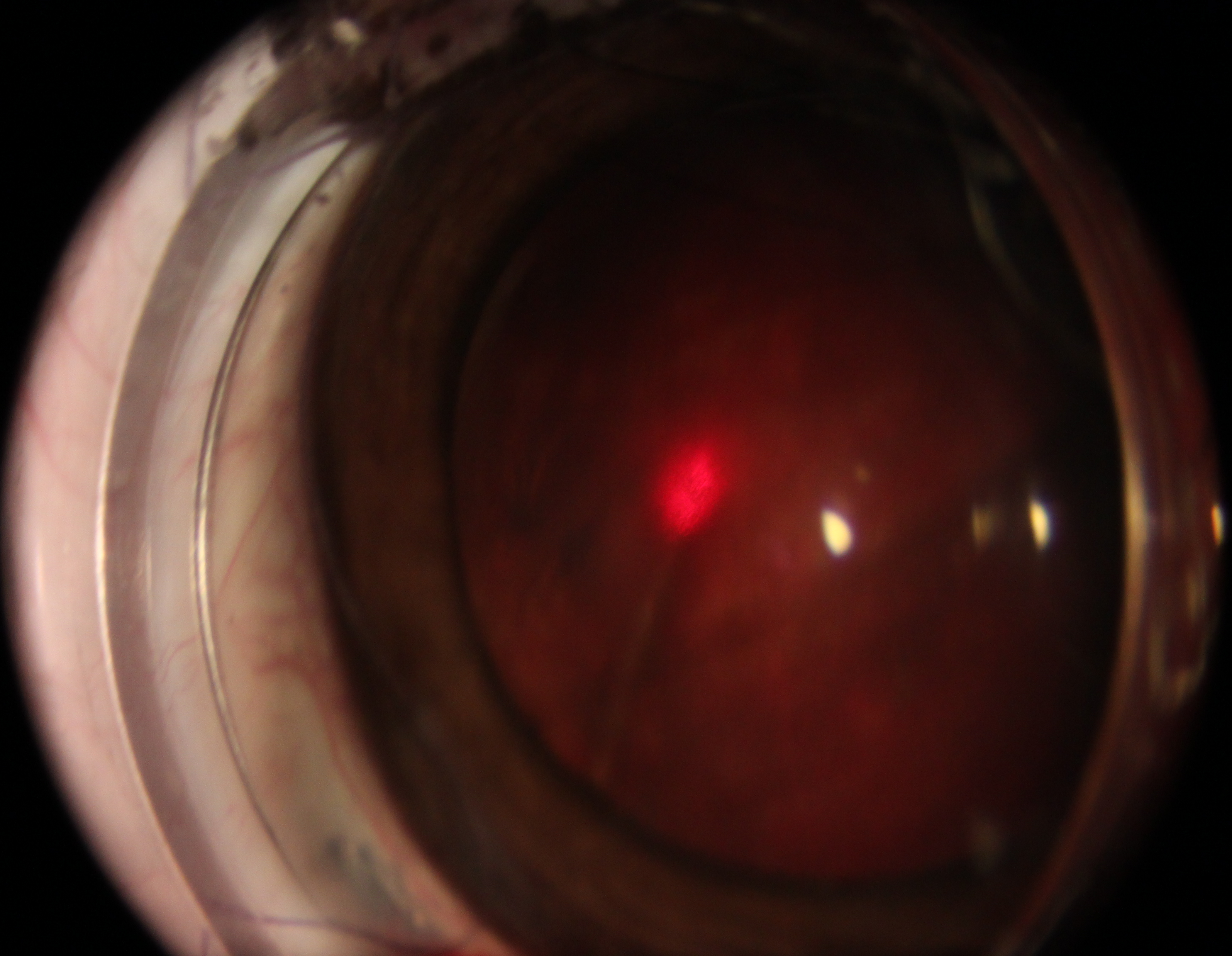

Supplement: Figure S1 — Illuminated microcatheter tip in the posterior suprachoroidal space, visualized by indirect ophthalmoscopy. (TIF) [file pone.0017140.s001.tif]

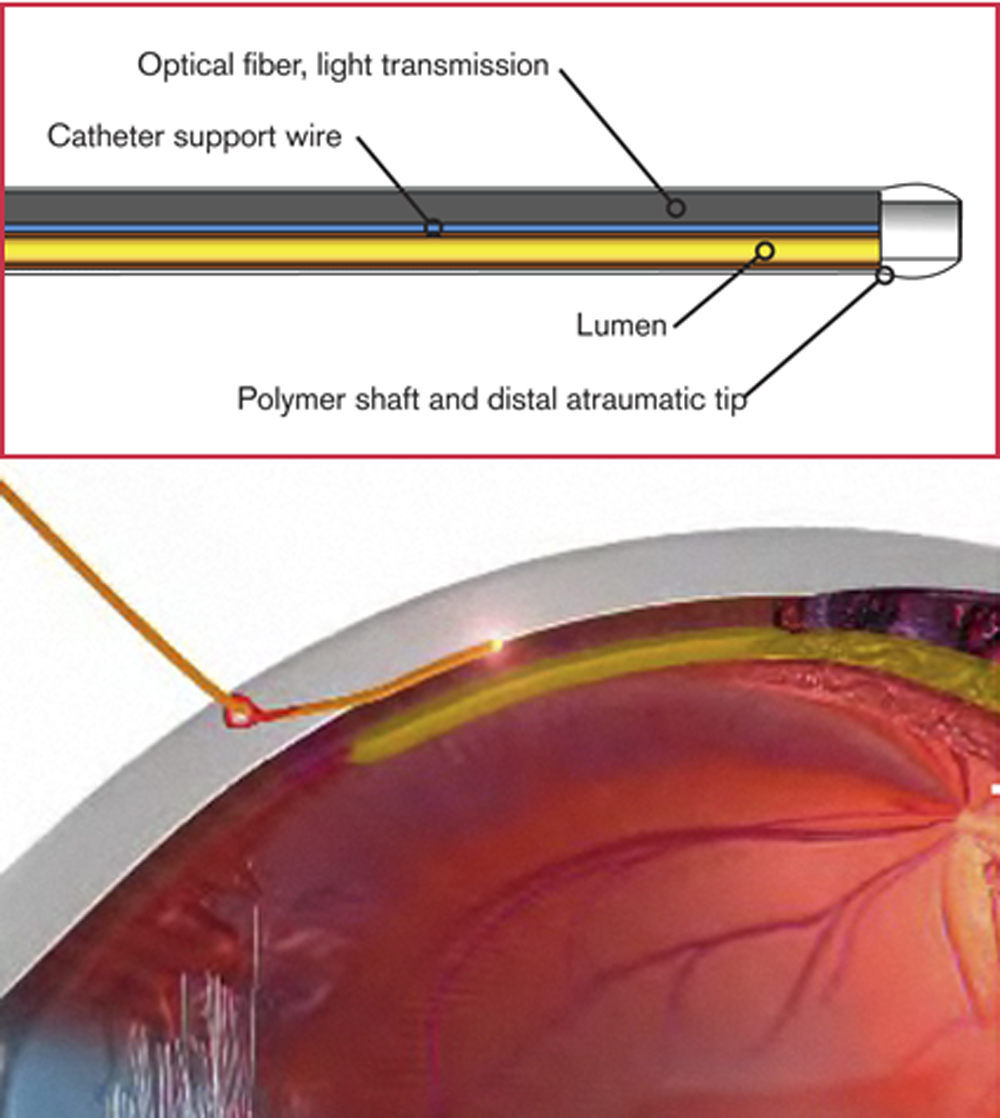

Supplement: Figure S2 — Schematic of iScience 250A microcannula (top) and introduction of the microcatheter through the sclerotomy into the suprachoroidal space and advanced towards the optic disc (bottom). Photographs courtesy of iScience Interventional. (TIF) [file pone.0017140.s002.tif]
